# Myricetin Induces Autophagy and Cell Cycle Arrest of HCC by Inhibiting MARCH1-Regulated Stat3 and p38 MAPK Signaling Pathways

**DOI:** 10.3389/fphar.2021.709526

**Published:** 2021-10-18

**Authors:** Wei Yang, Jiaqi Su, Mingjing Li, Tiantian Li, Xu Wang, Mingdong Zhao, Xuemei Hu

**Affiliations:** ^1^ Department of Imaging, Binzhou Medical University, Yantai, China; ^2^ Department of Chinese Medicine Prescription, Binzhou Medical University, Yantai, China; ^3^ Department of Immunology, Medical School, Qingdao University, Qingdao, China; ^4^ Department of Nuclear Medicine, Binzhou Medical University, Binzhou, China; ^5^ Department of Immunology, Binzhou Medical University, Yantai, China

**Keywords:** HCC, MARCH1, autophagy, proliferation, p38 MAPK, Stat3, cell cycle

## Abstract

Myricetin is a type of natural flavonol known for its anticancer activity. However, the molecular mechanism of myricetin in anti-hepatocellular carcinoma (HCC) is not well defined. Previous studies indicated that downregulation of membrane-associated RING-CH finger protein 1 (MARCH1) contributed to the treatment of a variety of cancers. Whether the anticancer property of myricetin is associated with MARCH1 expression remains to be investigated. This research explored the anti-HCC mechanism of myricetin. Our results indicate that myricetin induces autophagy and arrests cell cycle at the G2/M phase to suppress the proliferation of HCC cells by downregulating MARCH1. Myricetin reduces MARCH1 protein in Hep3B and HepG2 cells. Interestingly, myricetin upregulates the MARCH1 mRNA level in Hep3B cells but downregulates it in HepG2 cells. The knockdown of MARCH1 by siRNAs (small interfering RNAs) decreases the phosphorylated p38 MAPK (p-p38 MAPK) and Stat3 (p-Stat3), and inhibits HCC cell viability. Moreover, myricetin inhibits p38 MAPK and Stat3 signaling pathways by downregulating MARCH1 to repress HCC growth both *in vitro* and *in vivo*. Bafilomycin A1 (BafA1), an autophagy inhibitor, has synergetic effect with myricetin to inhibit HCC growth. Taken together, our results reveal that myricetin inhibits the proliferation of HCC cells by inhibiting MARCH1-regulated p38 MAPK and Stat3 signaling pathways. This research provides a new molecular mechanism for myricetin in anti-HCC and suggests that targeting MARCH1 could be a novel treatment strategy in developing anticancer therapeutics.

## 1 Introduction

Hepatocellular carcinoma (HCC) is the sixth commonly diagnosed cancer and the third leading cause of cancer-related deaths worldwide in 2020 (Sung et al., 2021). Liver cancer is responsible for 12.9% of cancer-related deaths and becomes the third cause of death in China (Feng et al., 2019). Because early diagnosis of HCC is difficult and its progression is rapid, the majority of HCC patients are diagnosed in the intermediate or advanced stage. The main treatment options for those patients are *trans*-arterial chemoembolization (TACE) and systemic therapy (El-Serag et al., 2008). Current existing chemotherapeutic drugs, such as sorafenib, often induce drug resistance and have multiple toxicities (El-Serag et al., 2008; Li et al., 2015). There is no effective systemic therapy for patients with advanced HCC. Therefore, it is necessary to explore more efficient drugs with less side effects and low toxicity for HCC treatment.

In recent years, increasing attentions have been paid on the research of natural compounds for cancer treatment and prevention. By 2010, antitumor drugs, partly or completely originated from natural products, accounted for over 40% of all anticancer drugs approved worldwide (Newman and Cragg, 2012). The clinical application of natural products as antitumor drugs has become an irresistible trend. Myricetin is a kind of natural flavonol which exists in various plants, such as vegetables, fruits, and nuts (Harnly et al., 2006). Myricetin has a variety of biological activities, such as antioxidation, anti-inflammation, and analgesic effects (Semwal et al., 2016). In addition, there are some reports suggesting that myricetin has anticancer effects on several types of cancers, such as pancreatic (Phillips et al., 2011), ovarian (Xu et al., 2016), prostate (Ye et al., 2018), and liver cancers (Zhang et al., 2011). Studies suggest myricetin shows cytotoxicity at IC50 in colon cancer (HT-29), prostate cancer (DU145), and breast cancer (T47D) cells at about 55 μM, 55.5, and 46 μM, respectively (Ye et al., 2018; Ma et al., 2019; Soleimani and Sajedi, 2020). The plasma concentration of myricetin in rats reached C_max_ of 1.49 μg/ml at approximately 6.4 h followed by a steady decline up to 24 h (Dang et al., 2014; Yao et al., 2014). It was reported that MEK1 and Stat3 pathway proteins play an active role in the initiation and progression of tumors (Cowley et al., 1994; Laudisi et al., 2018). Previous studies demonstrated that myricetin could directly interact with MEK1 and Jak-Stat3 (Lee et al., 2007; Kumamoto et al., 2009). However, the underlying molecular mechanism of myricetin in anti-HCC is unclear.

MARCH1 is a member of the membrane-anchored E3 ubiquitin ligases. Previously, the function of MARCH1 had been studied mainly in the immune system (Matsuki et al., 2007; Baravalle et al., 2011). Some studies reported that MARCH1 was overexpressed in ovarian and colorectal cancers, and knockdown of MARCH1 could inhibit the progression and development of cancer (Meng et al., 2016; Wang et al., 2021). Furthermore, studies in our lab found that MARCH1 was overexpressed in liver cancer (Xie et al., 2019a), and downregulation of MARCH1 induced by drugs such as secalonic acid-F and resveratrol could suppress HCC development (Xie et al., 2019b; Dai et al., 2020). Thus, we speculate that MARCH1 could be a promising antitumor therapeutic target.

By far, mounting evidence suggested that the effect of chemotherapeutic drugs on tumor is not limited to inhibiting biological functions such as cell cycle and proliferation but also on cell autophagy (Zhang et al., 2011; Chang et al., 2019). Autophagy is a highly conserved cellular recycling process in eukaryotes. In this process, cellular proteins, cytoplasmic organelles, and macromolecules are degraded in autolysosome. Degradation products from autolysosome are recovered for cell survival and maintenance of homeostasis (Yang and Klionsky, 2009). The signaling pathways that regulate autophagy include AMPK-LKB1 and PI3K/Akt/mTOR pathways and others (Casimiro et al., 2017; Xu et al., 2020). Accumulating studies revealed that p38 MAPK and Stat3 are involved in the regulation of autophagy (Ye et al., 2011; You et al., 2015). Simultaneously, p38 MAPK and Stat3 have oncogenic roles in the pathogenesis of cancer cells (Chai et al., 2016; Martínez-Limón et al., 2020). In this study, we explored the function of MARCH1 in the anti-HCC effect of myricetin both *in vitro* and *in vivo*. We also investigated the effect of myricetin on autophagy in HCC cells and its involvement of p38 MAPK and Stat3 signaling pathways.

## Materials and Methods

### Cell Culture

Hep3B and HepG2 human hepatocyte cancer cells were gained from Cell Lines Bank, Chinese Academy of Science (Shanghai, China). Cells were cultured in Dulbecco’s modified Eagle’s medium (DMEM) with high glucose (Hyclone, Logan, UT, United States); supplemented with 5% fetal calf serum (FBS) (Gibco Waltham, MA, United States),100 U/ml penicilin, and 100 μg/ml streptomycin (Solarbio, Beijing, China); and cultured at 37°C in a 5% CO_2_ humid atmosphere.

### Reagents and Antibodies

Myricetin (#HY-15097) was purchased from MedChemExpress (New Jersey, United States), dissolved in dimethyl sulfoxide (DMSO, #D8370, Solarbio, Beijing, China) at a concentration of 100 mM, and stored at −20 °C. MG132 (#HY-13259) and BafA1 (#HY-100558) were also bought from MedChemExpress and dissolved in DMSO. All antibodies were as follows: anti-Bcl-2 (#12789-1-AP), Ki-67 (#27309-1-AP), Stat3 (#10253-2-AP), LC3 (#14600-1-AP), P62 (#18420-1-AP), CyclinB1 (#55004-1-AP), CyclinD1 (#26939-1-AP), GAPDH (#10494-1-AP), and peroxidase-conjugated AffiniPure goat anti-rabbit IgG (H + L) (#SA00001-2) (Proteintech Group, Chicago, IL, United States); MARCH1 (#bs-9335R, Bioss, Beijing, China); p-Stat3 (#ab32143), p38 MAPK (#ab170099) (abcam, Cambridge, United Kingdom); p-p38 MAPK (#11581, Singalway Antibody, Maryland, United States); and MARCH1 (#YT2642, Immunoway, Newark, United States).

### Cell Transfection

All siRNAs and plasmids (pEX-3 and pEX-3–3×flag-MARCH1) were products of GenePharma (Shanghai, China). The sequences are as follows:

nontarget siRNA:

sense: 5′-UUC​UCC​GAA​CGU​GUC​ACG​UTT-3′;

antisense: 5′-ACG​UGA​CAC​GUU​CGG​AGA​ATT-3′.

MARCH1 siRNA1:

sense: 5′-CAG​GAG​GUC​UUG​UCU​UCA​UTT-3′;

antisense: 5′-AUG​AAG​ACA​AGA​CCU​CCU​GTT-3′.

MARCH1 siRNA2:

sense: 5′-GGU​AGU​GCC​UGU​ACC​ACA​ATT-3′;

antisense: 5′-UUG​UGG​UAC​AGG​CAC​UAC​CTT-3′.

SiRNAs or plasmids were dissolved in DEPC-treated water. Cells were seeded in a 6-well plate and grown up to 50% (for siRNAs transfection) or 90% (for plasmids transfection) confluence after 24 h. 2.6 μg siRNAs or 3.0 μg plasmids and 5 μl Lipofectamine 2000 (Invitrogen, Carlsbad, CA, United States) were separately diluted in 100 μl DMEM/high-glucose medium without FBS. After 5 min, two mixtures were mixed together. The mixtures were instilled into a 6-well plate after incubating at room temperature for 20 min. The cells were cultured for 48 h and collected for Western blot analysis.

### qRT-PCR

Total RNAs were extracted using TRIZOL reagents. The primers of human GAPDH and MARCH1 were custom-synthesized products of Takara (Dalian, China):

GAPDH sequences:

F: 5′-GCA​CCG​TCA​AGG​CTG​AGA​AC-3′,

R: 5′-TGG​TGA​AGA​CGC​CAG​TGG​A-3'.

MARCH1 sequences:

F: 5′-CTG​CTG​TGA​GCT​CTG​CAA​GTA​TGA-3′,

R: 5′-TAC​GTG​GAA​TGT​GAC​AGA​GCA​GAA-3'.

cDNAs were prepared using a Reverse Transcriptase Kit (#AG11711, ACCURATE BIOLOGY, Changsha, China). The cDNA amplification reactions were carried out using a SYBR^®^ Green Premix Pro Taq HS qPCR Kit II (#AG11702, ACCURATE BIOLOGY, Changsha, China). Quantitative real-time PCR analysis reactions were performed on LightCycler^®^ 96 (Roche, Switzerland). The PCR products were used for agarose gel electrophoresis. Grayscale analysis was performed by ImageJ.

### Western Blot

The collection of proteins was accomplished as follows: cells or tissues were put in a precooling RIPA lysis buffer (#P0013B, Beyotime, Shanghai, China), incubated on ice for 40 min, centrifuged at 12,000 rpm for 20 min. The protein supernatant was transferred into new tubes. Protein concentrations were detected by the BCA Protein Assay Kit (#PC0020, Solarbio, Beijing, China) to acquire protein concentrations. Lysate was blended in 5× SDS-PAGE loading buffer and then boiled for 8 min at 99°C. About 20 μg protein were diffused in 5% concentration gels and 12% separation gels. Then, the proteins on gels were transferred onto the polyvinylidene difluoride membrane (PVDF) (#ISEQ00010, Immobilon®-PSQ PVDF 0.2 μm, Ireland). Membranes were blocked in 5% skim milk for 2.5 h at room temperature and incubated with primary antibodies overnight at 4°C. Membranes were washed for 40 min with TBS-T. Then, the membranes were incubated with secondary antibodies for 40 min at 37°C. In the end, with abundant supersensitive ECL luminescent solution, protein imaging was performed by ChemiDocTM XRS+ (BIO-RAD, Hercules, CA, United States).

### Cell Proliferation Assay

CCK-8 (Cell Counting Kit-8) assay was carried out as follows: 5,000 cells were seeded in each well of the 96-well plates and cultured overnight at 37°C. After treating with myricetin for 24 h, the cells were incubated with CCK-8 reagents (Biosharp, Beijing, China) for 1 h at 37°C. The light absorbance at 450 nm was measured by a microplate reader (SpectraMax M2, Molecular Devices, Shanghai, China).

The EdU (5-Ethynyl-2′-Deoxyuridine) proliferation assay was determined by using EdU detection kits (#C10310-1, Ribobio, Guangzhou, China) according to the manufacturer’s instruction. 5,000 cells were seeded in 96-well plates per well and cultured overnight. The cells were then treated with myricetin for 24 h. Then, the cells were stained with 50 μM EdU medium for 2 h, immobilized with 4% polyoxymethylene for 30 min, treated with 2 mg/ml glycine for 5 min, and permeated by 0.5% TritonX-100 for 10 min. After that, the cells were mixed with Apollo 567 for 30 min at room temperature in the dark and permeated again by 0.5% TritonX-100 for 20 min. Last, cells were maintained in Hoechst 33,342 for 30 min at room temperature in the dark. Cells were detected by the fluorescence microscope (Olympus TL4 photomicroscope, Japan). Proliferative cell nuclei were stained red fluorescence, and all cell nuclei showed blue fluorescence. The cell proliferation rate was analyzed by ImageJ.

### Cell Cycle Analysis

The distribution of cells at each cycle was examined by the flow cytometer. The cells were collected after treating myricetin for 24 h, and then maintained in precooled 75% ethanol overnight at 4°C. Cells were washed and resuspended using phosphate buffer solution (PBS). RNase (Sikh Association of Baltimore) was added to the cells, and the cells were incubated for 30 min at 37°C. Then, the cells were mixed with propidium iodide (PI, Sikh Association of Baltimore) for 30 min at 4°C. Fluorescence intensity was measured within one hour.

### Immunofluorescence Analysis

1×10^4^ cells were seeded on culture dishes. The cells were treated with myricetin for 24 h and washed three times with PBS. Then, cells were immobilized with cold methanol for 15 min, permeated with 1% TritonX-100 for 10 min, and blocked with goat serum for 30 min. Subsequently, cells were incubated with LC3 antibody (1:200 dilution) at room temperature for 1 h and integrated with DyLight 488 goat anti-rabbit secondary antibody (#A23220, Abbkine, Wuhan, China; 1:300 dilution). The nuclei were stained with 4′,6-diamidino-2-phenylindole dihydrochloride (DAPI, #C0065, Solarbio, Beijing, China; 1:500 dilution) for 5 min. Finally, cells were observed by a LSM880 laser scanning confocal microscope (ZEISS, Germany) system.

### Animals and Treatment

4- to 5-week-old female BALB/c nude mice were obtained from SiPeiFu company (Beijing, China) and housed in the specific pathogen-free (SPF) animal laboratory in Binzhou Medical University. The animal study was reviewed and approved by the Animal Experimental Ethics Committee of Binzhou Medical University. The mice were allowed to adapt to the new environment for 5 days. Approximately 1×10^7^ HepG2 cells, resuspended in PBS, were subcutaneously injected into the dorsal region near the right hind leg of the nude mice. When the tumor size reached about 100 mm^3^, the mice were randomly assigned to control and treatment groups with eight mice in each group. Hereafter, the mice were daily administered with corn oil or myricetin (dissolved in corn oil, 25 mg/kg body weight) by gavage. The weight of mice and tumor volume were measured once every 2 days during the therapeutic session of 25 days. And the tumor volume was calculated with the following formula: V = A × B^2^× 0.5, where A and B represent length and width, respectively. Blinding was applied during acquisition of tumor volume when possible. After 25 days, the mice were executed. Tumors were taken out, weighed, fixed by 4% paraformaldehyde, or cryopreserved for next experiments, such as hematoxylin–eosin staining, immunohistochemical analysis, and Western blot analysis.

### Histology and Immunohistochemistry

Tumor tissues were fixed in 4% paraformaldehyde, processed through a series of dehydration steps, and embedded into paraffin blocks. Paraffin-embedded tissues were cut into 4 μm slices and adhered on adhesive slides. Tissue sections were stained with hematoxylin–eosin. For immunohistochemistry, sections were de-waxed, antigen-repaired, and blocked for nonspecific reactions. Then, the sections were incubated with anti-MARCH1 (1:200, #YT2642, Immunoway, Newark, United States) or anti–Ki-67 (1:200) antibody for overnight at 4 °C and then incubated with secondary antibody (#PV-6000, ZSGB-BIO, Beijing, China) for 30 min at 37°C. Diaminobenzidine (#ZLI-9018, ZSGB-BIO, Beijing, China) was used for color development. The sections were imaged with a light microscope (Leica Microsystems, Wetzlar, Germany). Quantification of section staining was performed by Image-Pro Plus 6.0.

### Statistical Analysis

Data derived from at least three experiments are reported as the means ± S.D. Differences between two or more groups were subjected to two-tailed Student’s *t* test or ANOVA. Statistical analyses were conducted using Prism 7 (GraphPad Software Inc., San Diego, United States). *p* < 0.05 was considered statistically significant.

## Results

### Myricetin Suppresses HCC Cell Growth by Down-Regulating MARCH1 Expression

**FIGURE 1:**
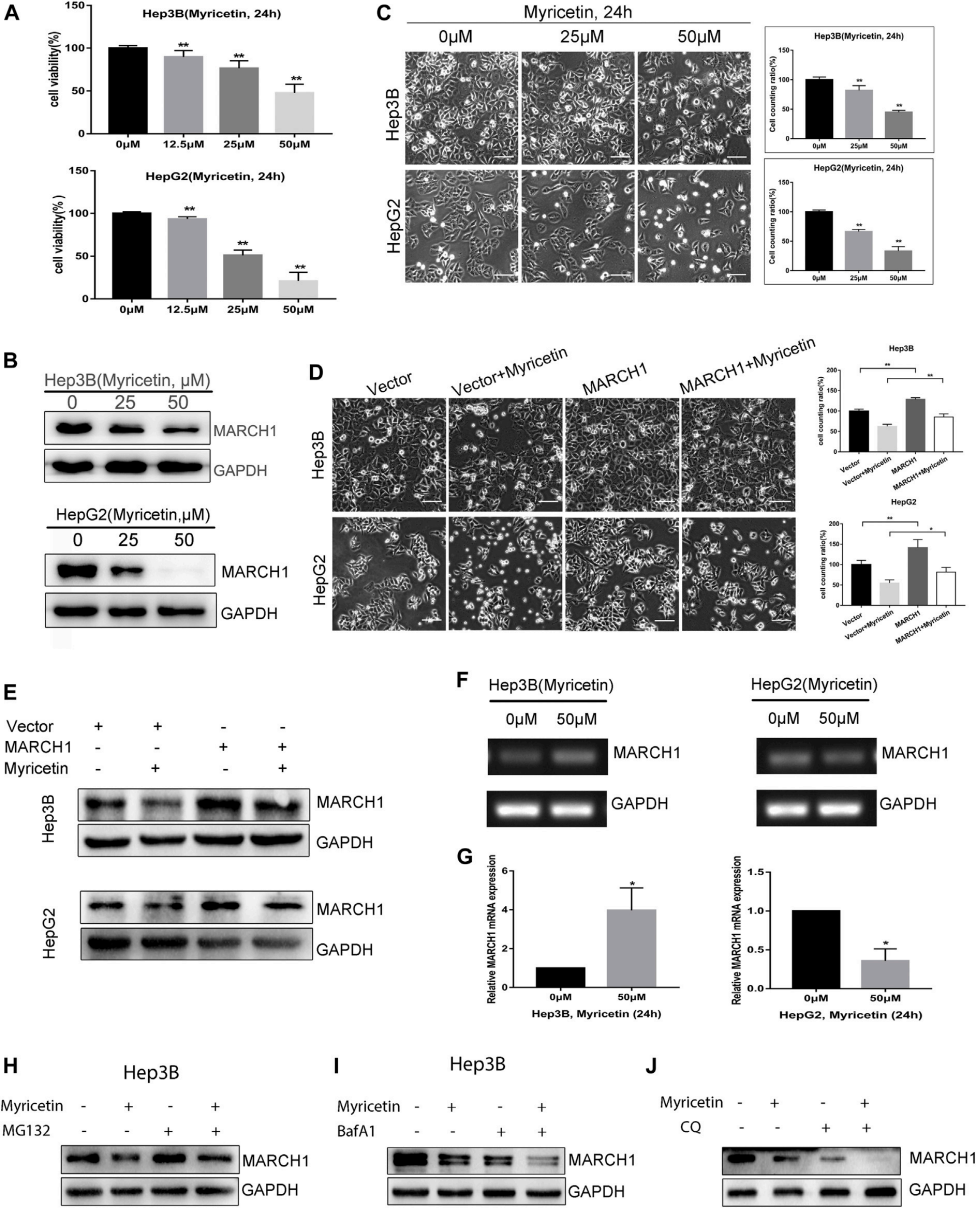
**(A)** The viability of HCC cells assessed by CCK-8 reagent after myricetin treatment for 24 h. **(B)** MARCH1 expression in Hep3B and HepG2 cells treated with myricetin for 24 h detected by Western blot. **(C)** Images showing the viability of HCC cells incubated with myricetin for 24 h. Surviving cells were statistically and quantitatively analyzed (bar: 100 μm). **(D)** HCC cells, transfected empty or MARCH1 plasmids, were treated with 50 μM myricetin for 24 h. Cells state as shown, bar: 100 μm. **(E)** MARCH1 expression was detected by Western blot. **(F)** MARCH1 mRNA in HCC cells treated with 50 μM myricetin for 24 h measured using agarose gel electrophoresis. Representative images are shown. GAPDH is used as the loading control. **(G)** The mRNA level of MARCH1 of HCC cells incubated with myricetin for 24 h analyzed by qRT-PCR. **(H)** Hep3B cells pretreated with 0.25 μM MG132 for 4 h and with 50 μM myricetin for 24 h. MARCH1 expression was monitored by Western blot. **(I)** Hep3B cells pretreated with 100 nM BafA1 for 8 h and then treated with 50 μM myricetin for 24 h. MARCH1 expression was analyzed. **(J)** Hep3B cells pretreated with 40 μM CQ for 4 h and then treated with 50 μM myricetin for 24 h. MARCH1 expression was analyzed. All data are from the results of the three experiments, presented as the means ± SD. * represents *p* < 0.05, ** represents *p* < 0.01.

HCC cells treated with myricetin were incubated with the CCK-8 reagent. Results showed that the viability of HCC cells treated with myricetin was obviously declined in a dose-dependent manner (Figure 1A). IC50 of myricetin for Hep3B and HepG2 was 48.473 and 28.147 μM, respectively. To investigate whether MARCH1 is involved in the anti-HCC effect of myricetin, HCC cells were treated with different doses of myricetin for 24 h. The expression of MARCH1 in Hep3B and HepG2 cells decreased as detected by Western blot analysis (Figure 1B). And the number of living HCC cells exposed to myricetin significantly descended, especially at the concentration of 50 μΜ (Figure 1C). To verify that the anti-HCC effect of myricetin is mediated by downregulating MARCH1, HCC cells transfected with MARCH1 plasmid were treated with 50 μΜ myricetin. Results showed that the overexpression of MARCH1 partially offset the antitumor effect of myricetin (Figure 1D), and the overexpression of MARCH1 partially saved MARCH1 downregulation induced by myricetin (Figure 1E). Interestingly, we found that the expression of MARCH1 mRNA was different in Hep3B and HepG2 cells treated with myricetin. Myricetin slightly increased the mRNA level of MARCH1 in Hep3B cells, but obviously decreased the mRNA level of MARCH1 in HepG2 cells in agarose gel electrophoresis and qRT-PCR analysis (Figures 1F,G). MG132 (a proteasome inhibitor) and BafA1 (a lysosomal or autophagy inhibitor (Yoshimori et al., 1991)) were used to explore if MARCH1 protein down-regulation in Hep3B cells is *via* proteasome and lysosomal pathway. The results showed that inhibition of proteasome or lysosome could not rescue the decrease of MARCH1 protein level caused by myricetin (Figures 1H,I). Chloroquine (CQ), also an autophagy inhibitor, was cotreated with myricetin on Hep3B cells. MARCH1 expression was detected by Western blot analysis. As shown, CQ did not rescue the MARCH1 downregulation caused by myricetin. Inversely, CQ or BafA1 with myricetin cotreatment further decreased MARCH1 expression. These results demonstrated that myricetin suppressed the growth capacity of HCC cells, and myricetin downregulates MARCH1 protein in HCC cells to inhibit HCC growth.

### Myricetin Induces Autophagy in HCC Cells in a MARCH1-Dependent Manner and Synergistically Inhibits HCC Growth With BafA1

**FIGURE 2:**
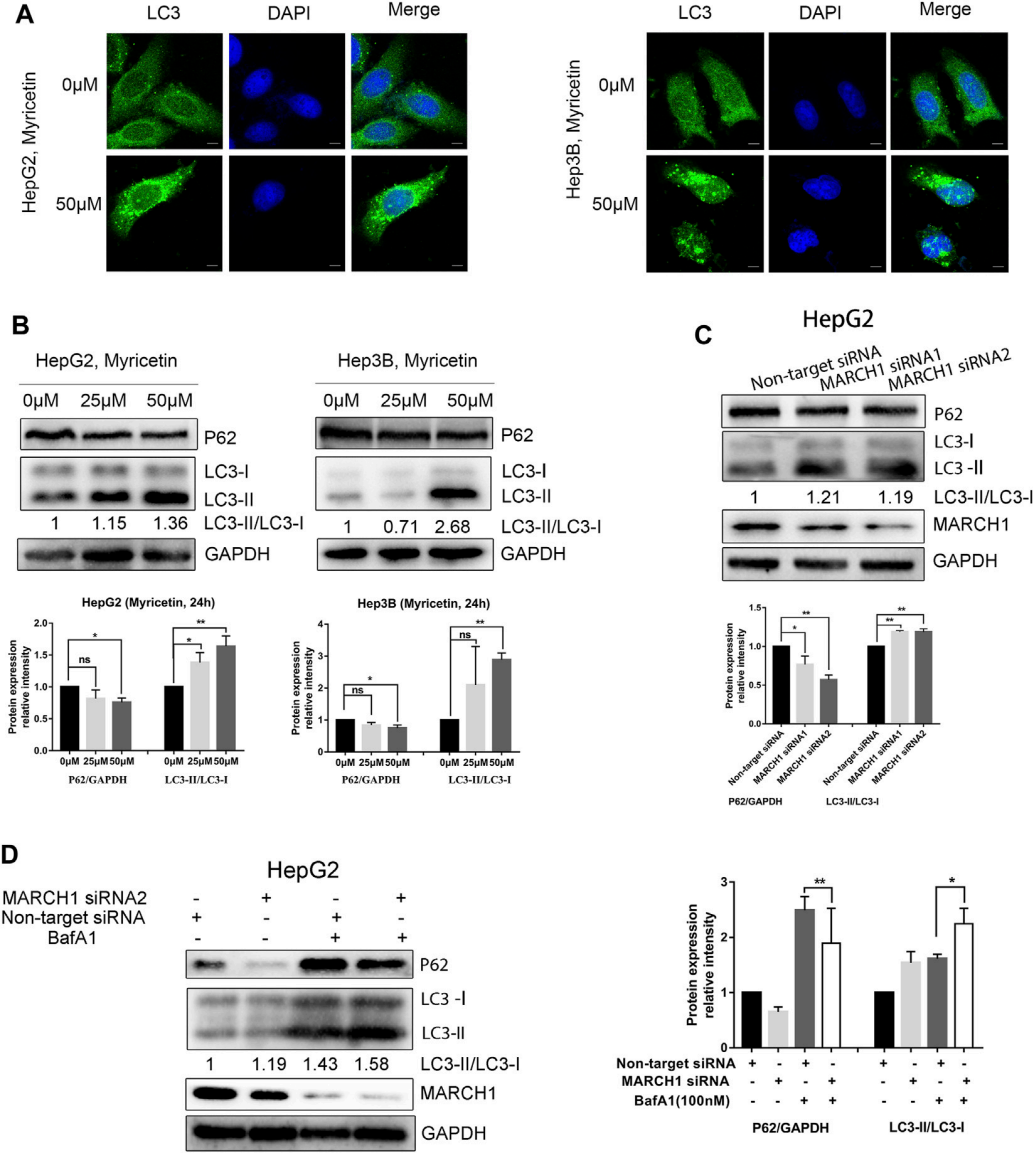
**Myricetin induces autophagy of HCC cells by downregulating MARCH1. (A)** Immunofluorescence staining used to detect the endogenous LC3 punta to assess autophagy activity of HCC cells treated with myricetin, bar: 10 μm. **(B)** The expression of autophagy marker proteins LC3 and P62 detected by immunoblotting in HCC cells treated with indicated doses of myricetin for 24 h **(C)** LC3 and P62 expression in HepG2 cells transfected with or without siRNA MARCH1 analyzed by Western blot. **(D)** HepG2 cells, transfected with siRNAs and mixed with or without 100 nM BafA1, were incubated for 48 h. The expression of LC3 and P62 measured by immunoblotting. Band intensity determined by ImageJ. All data are from the results of the three experiments, presented as the means ± SD. * represents *p* < 0.05, ** represents *p* < 0.01.

**FIGURE 3:**
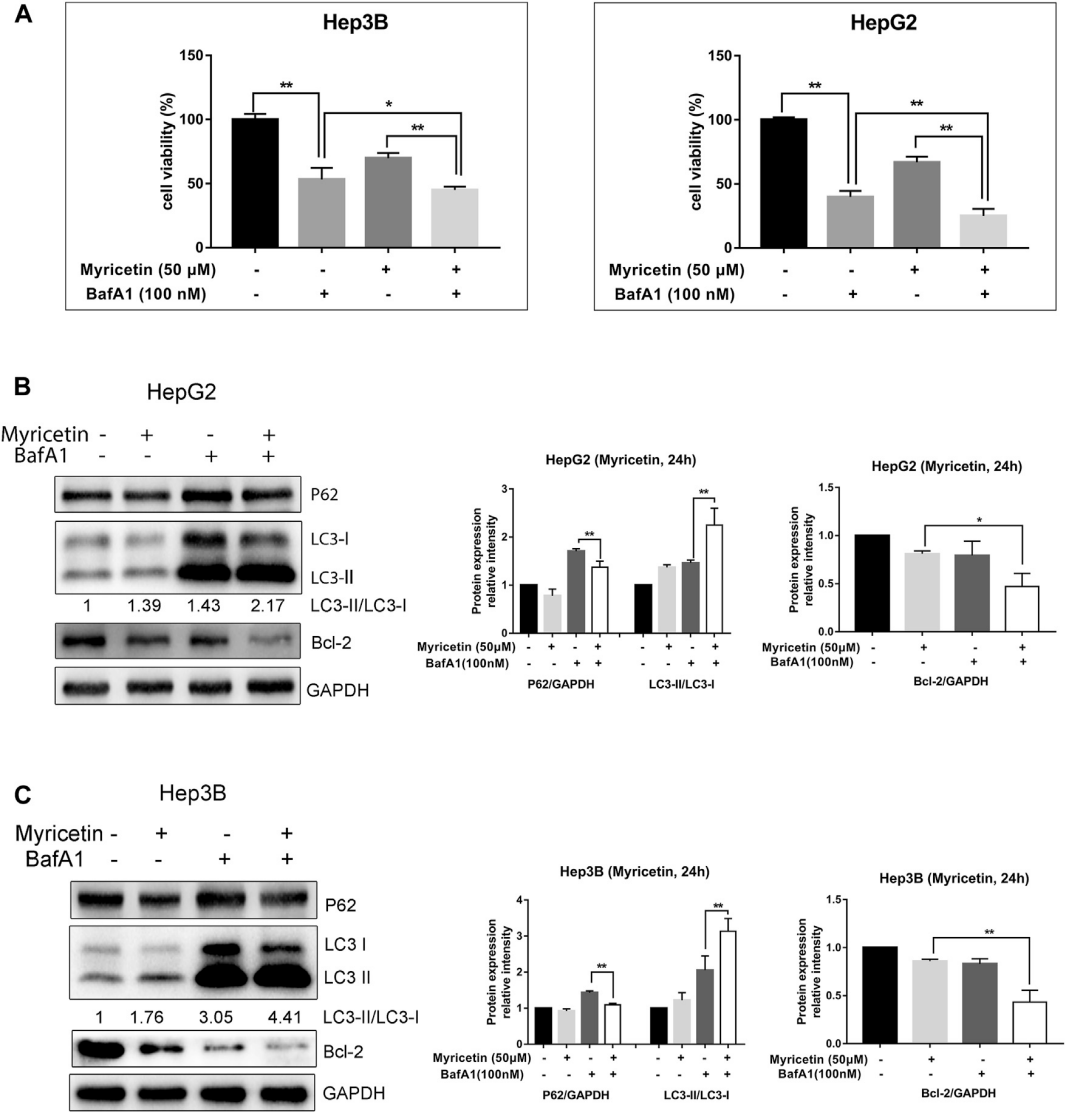
**Myricetin synergistically inhibits HCC growth with BafA1. (A)** The viability of HCC cells was assessed by CCK-8 reagent under the effect of combination of 50 μM myricetin and 100 nM BafA1. **(B)** HepG2 cells were treated with 100 nM BafA1 (pre-treated for 4 h) and 50 μM myricetin (for 24 h), followed by Western blot to analyze LC3, P62 and Bcl-2 protein. **(C)** LC3 and P62 expressions in Hep3B cells detected when cells were treated with myricetin and BafA1. Blots analyzed by ImageJ. All data are from results of three experiments, presented as the means ± SD. * represents *p* < 0.05, ** represents *p* < 0.01.

Although BafA1 pretreated HCC cells could not restore the decrease of the MARCH1 protein level caused by myricetin treatment, BafA1 could enhance the antiproliferative effect of myricetin in HCC cells. HCC cells, with or without the pretreatment of 100 nM BafA1 for 4 h, were treated with myricetin (50 μM) for 24 h. The cell viability was weakened after the cells were treated with myricetin in combination with BafA1, compared to the cells treated with myricetin or BafA1 alone (Figure 3A). BafA1 is a lysosomal inhibitor and also an autophagy inhibitor (Greene et al., 2012). Therefore, BafA1 enhanced the sensitivity of HCC cells to myricetin might be related to autophagy. To examine the autophagic activity of HCC cells in the presence of myricetin, immunofluorescence assay was performed. The results showed that the quantity of endogenous LC3-positive puncta increases in HepG2 and Hep3B cells (Figure 2A). The autophagic activity was further validated by measuring the protein expression of LC3 and P62 in HCC cells using Western blot analysis. The results demonstrated that myricetin increases the LC3-II/LC3-I ratio and decreases P62 levels in both HCC cell lines (Figure 2B). Also, the LC3-II/LC3-I ratio was increased and P62 expression was reduced in HepG2 cells when MARCH1 expression was downregulated by siRNAs (Figure 2C). After BafA1 was used to block the process of autophagy, the LC3-II/LC3-I ratio remained increased and P62 expression was decreased (Figure 2D), suggesting that MARCH1 downregulation could induce HCC cell autophagy. Western blot analyzed the expression of autophagic- and apoptotic-related proteins after BafA1 and myricetin cotreated HCC cells. Results showed that the LC3-II/LC3-I ratio was increased and the expression of P62 was slightly declined in HCC cells (Figures 3B,C). In addition, proapoptotic protein Bcl-2 was significantly down-regulated in HCC cells after being treated with myricetin and BafA1 (Figures 3B,C). These results indicated that myricetin induced autophagy in a MARCH1-dependent manner in HCC cells. And myricetin worked synergistically with BafA1 to suppress HCC growth.

### Myricetin Arrests Cell Cycle at the G2/M Phase to Reduce HCC Proliferation

**FIGURE 4:**
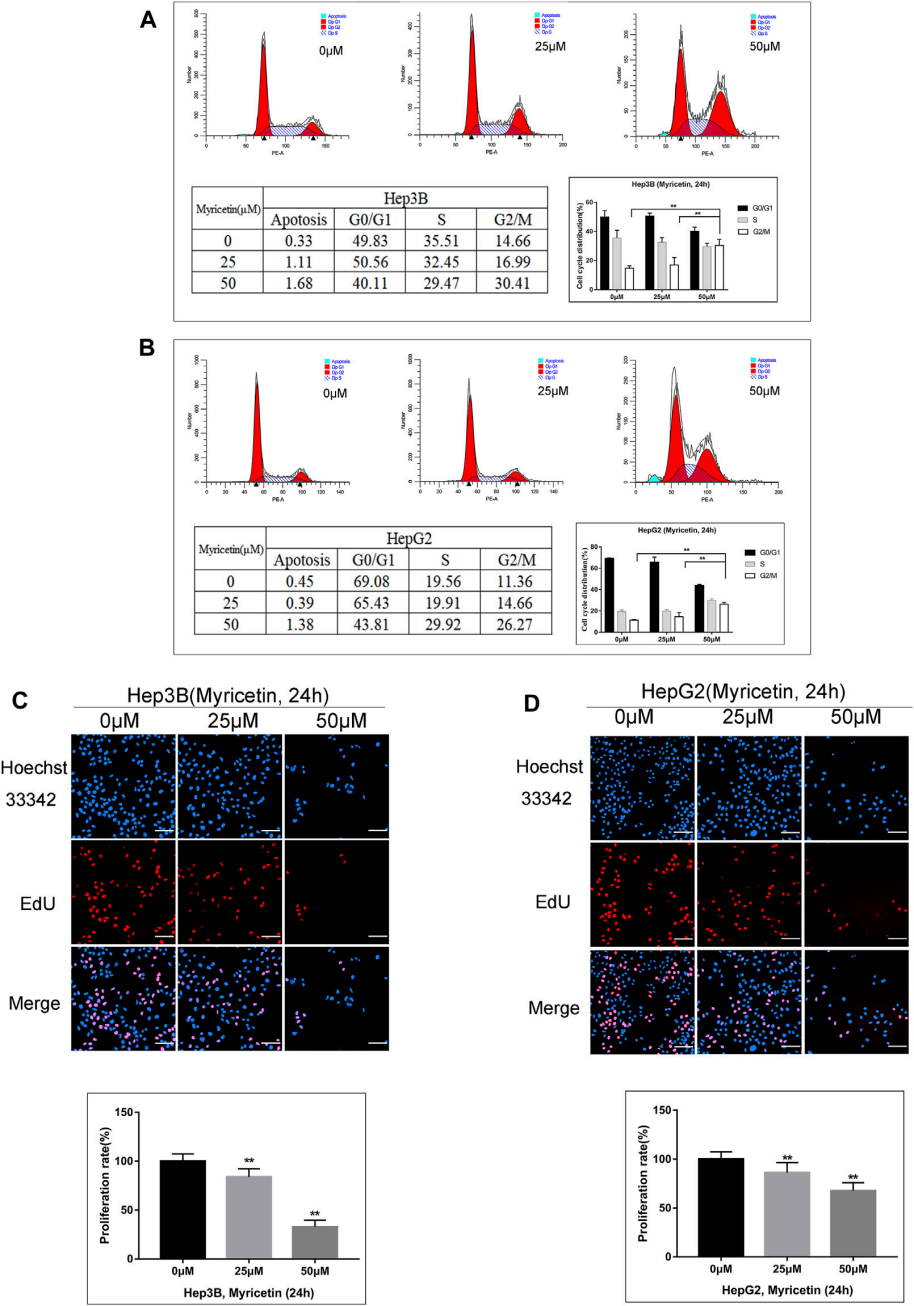
**Myricetin represses HCC proliferation and arrests cell cycle at G2/M phase in HCC cells. (A)** Flow cytometry on cell cycle distribution of HepG2 cells treated with specified doses of myricetin for 24 h. **(B)** Cell cycle distribution of Hep3B cells. **(C)** The representative images showed the state of Hep3B cells proliferation with indicated does of myricetin treatment. Proliferation cells were stained with EdU (red), bar: 100 μm. **(D)** The EdU assay was carried out to analyze proliferation of HepG2 cells treated with or without myricetin, bar: 100 μm. All data are from results of three experiments, presented as the means ± SD. * represents *p* < 0.05, ** represents *p* < 0.01.

The above results indicated that myricetin could inhibit HCC growth. To explore whether cell cycle arrest is related with myricetin treatment in HCC cells, flow cytometric analysis was performed. We found that the cell cycle was arrested at the G2/M phase in both Hep3B and HepG2 cells treated with myricetin; simultaneously, there was an obvious decrease of cell number in the G0/G1 phase (Figures 4A,B). The proliferation of HCC cells was evaluated using an EdU kit. The Hep3B and HepG2 cells were treated with myricetin for 24 h and processed according to the manufacturer’s instruction of the kit. Results showed that the proliferative nuclei (stained with EdU) were dramatically declined in a concentration-dependent manner when cells were exposed in myricetin (Figures 4C,D). These results indicated that myricetin suppressed HCC cell proliferation, most likely by blocking cell cycle at the G2/M phase.

### Myricetin Induces Autophagy and Cell Cycle Arrest of HCC Cells by Inhibiting MARCH1-Regulated Stat3 and p38 MAPK Signaling Pathways

**FIGURE 5:**
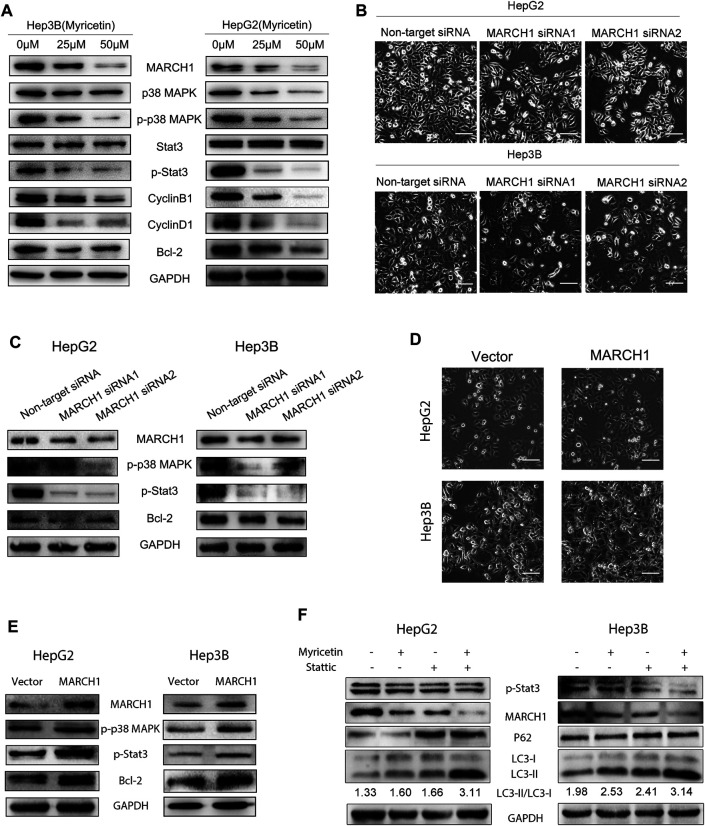
**Myricetin induces autophagy and cell cycle arrest of HCC cells via downregulating MARCH1-mediated p38 MAPK/Stat3 signaling pathway. (A)** HCC cells were treated with 0, 25, 50 μM myricetin for 24 h p38 MAPK and Stat3 signaling pathway proteins and cell cycle proteins were analyzed by Western blot. **(B)** Above images show the viability of HCC cells transfected with or without siRNA MARCH1 for 48 h (bar: 100 μm). **(C)** The p-p38 MAPK, p-Stat3, and Bcl-2 were detected by Western blot analysis after transfected siRNA MARCH1 or nontarget siRNA in HCC cells. **(D)** The growth ability of Hep3B and HepG2 cells transfected with or without MARCH1-plasmids is shown in the figure (bar: 100 μm). **(E)** HCC cells were transfected with MARCH1 plasmids for 48 h and conducted with Western blot to measure the expression of *p*-pretreatp38 MAPK, p-Stat3 and Bcl-2 proteins. **(F)** HCC cells were treated with a combination of 50 μM myricetin and 2.5 μM Stattic (for 4 h) for 24 h. Western blot was used to analyze the expression of MARCH1, LC3, P62.

The Stat3 and p38 MAPK signaling pathways play important roles in multiple biological activities of tumor cells such as proliferation, cell cycle, and autophagy. Western blot analysis was used to explore whether myricetin induces autophagy and cell cycle arrest of HCC cells through modulating the MARCH1-regulated p38 MAPK/Stat3 signaling pathway. It was found that the expression of p-p38 MAPK and p-Stat3 is declined in HCC cells with myricetin treatment (Figure 5A), and cell cycle proteins Bcl-2, CyclinB1, and CyclinD1 are also significantly reduced (Figure 5A). To identify whether MARCH1 is involved in the regulation of p38 MAPK and Stat3 signaling pathways by myricetin, MARCH1 was knocked down by siRNAs in HCC cells to detect the changes of expression of p38 MAPK and Stat3 and the viability of HCC cells. The results showed that MARCH1 knockdown inhibited the proliferation of HCC cells (Figure 5B), which was consistent with a previous report (Xie et al., 2019a). Loss of MARCH1 expression resulted in the downregulation of expression of p-p38 MAPK, p-Stat3, and Bcl-2 (Figure 5C), suggesting that MARCH1 could regulate p38 MAPK and Stat3 signaling pathways. We also verified the above results from the opposite direction by overexpressing MARCH1 in the HCC cells. After transfection of MARCH1 plasmids, not only the growth of HCC cells improved (Figure 5D) but the expression of p-p38 MAPK, p-Stat3, and Bcl-2 was also upregulated (Figure 5E). In addition, Stattic, a Stat3 inhibitor, was used to treat HCC cells combined with myricetin. Western blot results showed that Stattic increased P62 expression and the LC3-II/LC3-I ratio, and Stattic combined with myricetin further raised the LC3-II/LC3-I ratio. We also found that Stat3 inhibition could result in MARCH1 downregulation (Figure 5F). These results demonstrated that myricetin induced cell cycle arrest at the G2/M phase and autophagy of HCC cells to further suppress HCC growth by inhibiting MARCH1-regulated Stat3 and p38 MAPK signaling pathways.

### Myricetin Represses HCC Tumor Growth *In Vivo*

**FIGURE 6:**
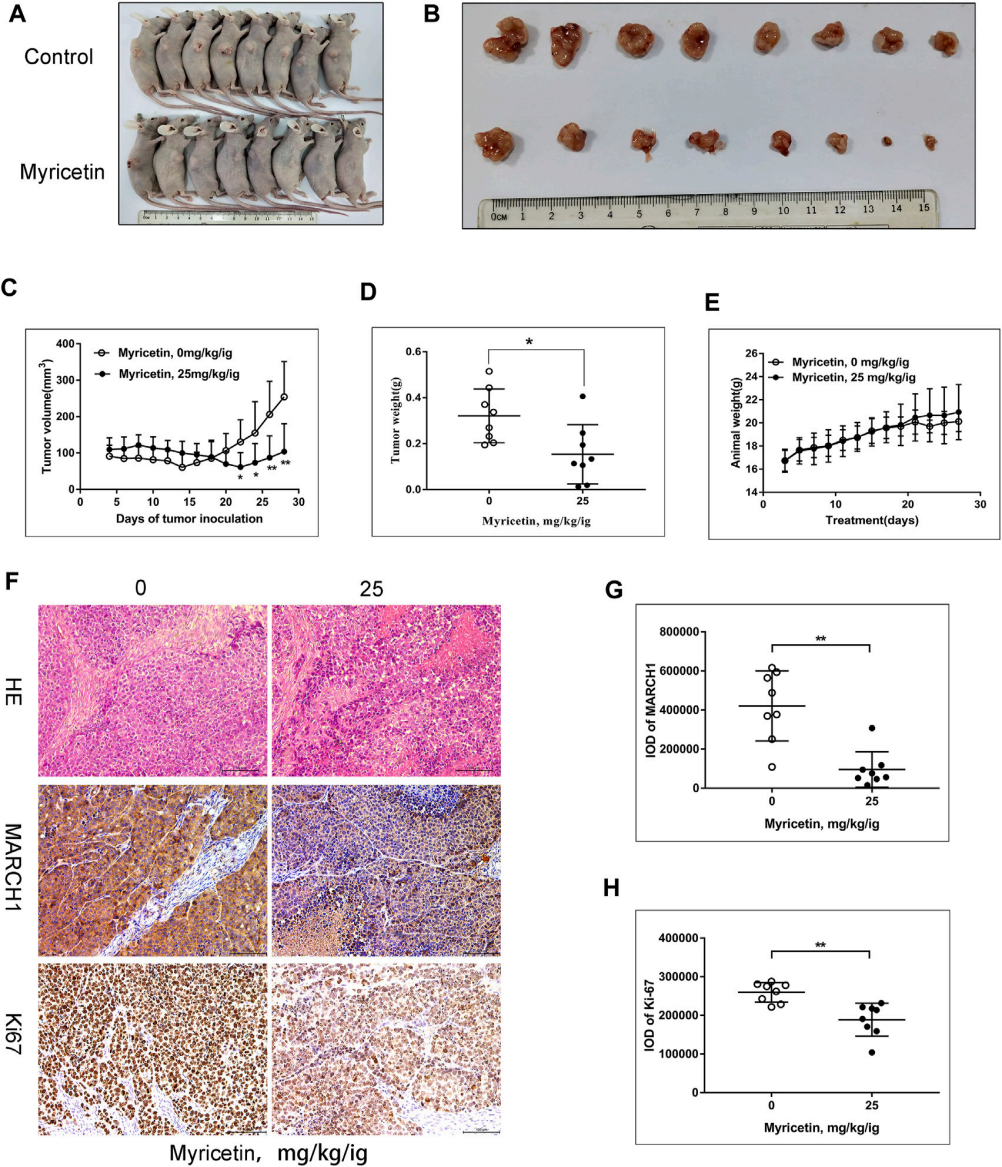
**Myricetin significantly reduces the growth of HCC xenograft tumor. (A)** Image of BALB/C nude mice with or without myricetin treatment. **(B)** Image of tumor tissue in myricetin treated or untreated group. **(C)** The tumor volume during myricetin treatment was plotted. **(D)** Tumor weight in myricetin treated and untreated groups.**(E)** Mice body weight during myricetin treatment. **(F)** Tumor tissue sections identified by hematoxylin–eosin staining and immunohistochemistry. **(G)** IOD of MARCH1 in immunohistochemistry. Three fields were randomly selected from each sample. **(H)** IOD of Ki-67 in immunohistochemistry. Five fields were randomly selected from each sample. All data are presented as the means ± SD. * represents *p* < 0.05, ** represents *p* < 0.01.

**FIGURE 7:**
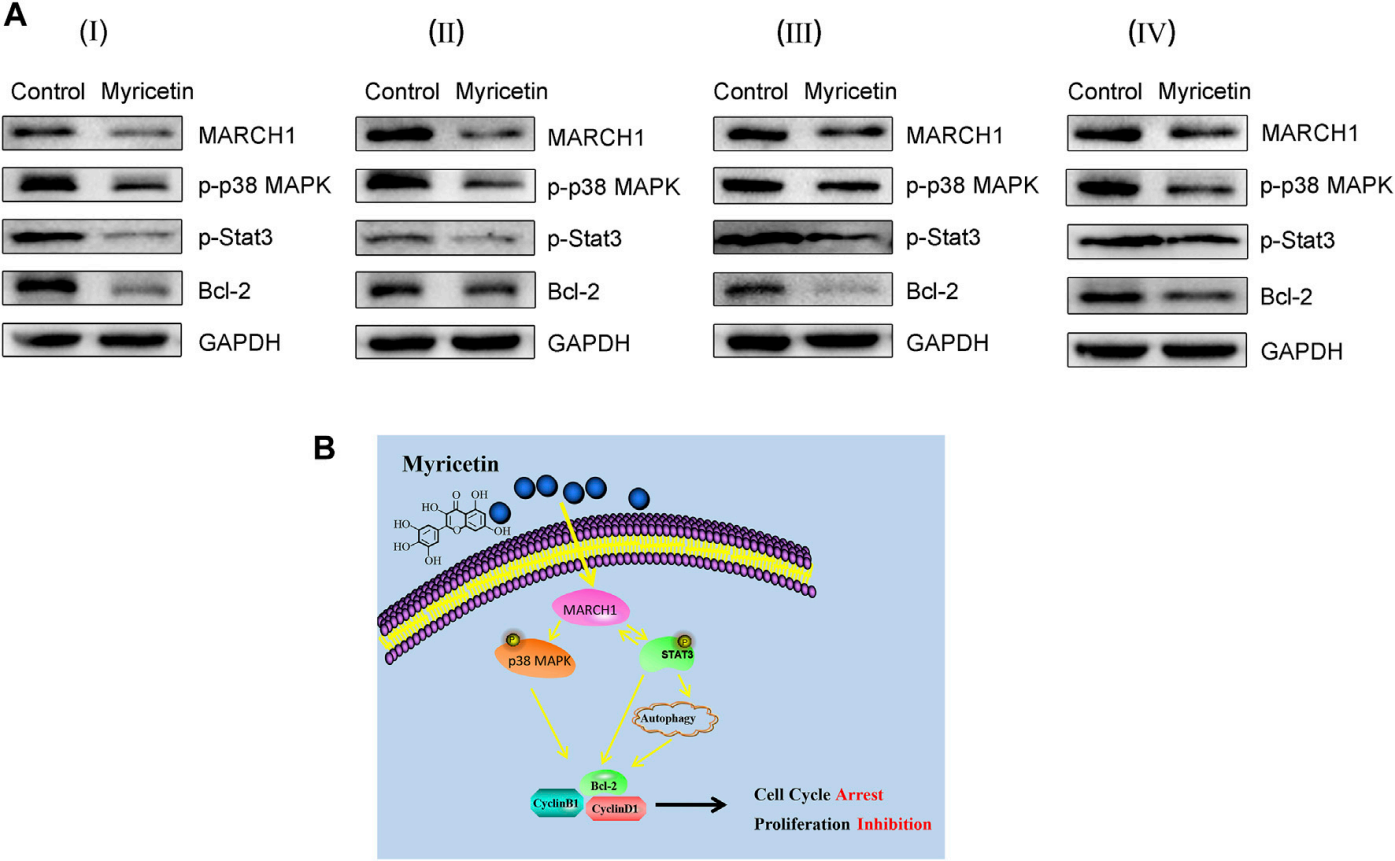
**Myricetin restricts HCC tumor growth by downregulating MARCH1-mediated of p38 MAPK/Stat3 pathway. (A)** The expression of MARCH1, p-p38 MAPK, p-Stat3, and Bcl-2 in tumor tissues detected by Western blot. **(B)** The mechanism model of myricetin in inducing HCC autophagy and cell cycle arrest to inhibit HCC proliferation.

To further assess the effect of myricetin on HCC tumor growth, female BALB/c nude mice were selected to establish the subcutaneous tumor xenograft model. Tumors were treated with myricetin (25 mg/kg/day) treatment for 25 days. The HCC tumor size of the treatment group reduced compared with that of the control group (Figures 6A–D). The body weight of mice was not changed between the myricetin treatment group and the control group (Figure 6E). Hematoxylin–eosin staining showed loose structure, more apoptotic cells, inflammatory cell infiltration, and necrosis of tumor cells in the section of tumor tissues treated with myricetin (Figure 6F). Immunohistochemical staining showed that the expression of MARCH1 and Ki-67 in tumor tissues of the myricetin treatment group was decreased (Figure 6F). MARCH1 and Ki-67 expression were quantitatively analyzed by Image-Pro Plus in at least three fields of each sample. The integrated optical density (IOD) of MARCH1 and Ki-67 showed a significant difference between the myricetin treatment group and control group (Figures 6G,H). The tumor tissue proteins were extracted, and Western blot was used to analyze the expression of MARCH1 and molecules in the p38 MAPK/Stat3 signaling pathway. The results showed that myricetin could downregulate MARCH1, p-p38 MAPK, p-Stat3, and Bcl-2 proteins *in vivo* (Figure 7A). These results indicated that myricetin inhibits the development of HCC by downregulating MARCH1/p38 MAPK/Stat3 signaling *in vivo*.

The molecular mechanism of myricetin in anti-HCC was displayed in Figure 7B. Myricetin induced autophagy and cell cycle arrest of HCC cells by inhibiting the MARCH1-regulated p38 MAPK/Stat3 signaling pathway to inhibit HCC cell proliferation.

## Discussion

Myricetin is a kind of natural flavonol and has been widely used in medicine, food, health-care products, and cosmetics approved by the Food and Drug Administration of the United States of America. Myricetin was involved in the regulation of various biological functions of tumors, such as proliferation, apoptosis, and cell cycle (Zhang et al., 2011; Xu et al., 2016; Ye et al., 2018). This study found that myricetin could induce cell autophagy and cell cycle arrest at the G2/M phase of HCC cells to inhibit cell proliferation. Myricetin combined with BafA1 synergstically suppressed HCC cells’ growth. The underlying mechanism of myricetin in anti-HCC proliferation might be associated with the downregulation of MARCH1, p-p38 MAPK, and p-Stat3 signaling pathway–related proteins.

MARCH1 was overexpressed in ovarian, colorectal, and HCC cancer cells. The downregulation MARCH1 was beneficial for the inhibition of cancer growth (Meng et al., 2016; Xie et al., 2019a; Wang et al., 2021). The results of the present study indicated that myricetin decreases the protein level of MARCH1 in HCC cells. However, myricetin inhibited the mRNA expression of MARCH1 in HepG2 cells but augmented it in Hep3B cells. This discrepancy might attribute to cell specificity. For example, HepG2 cell was p53 wild type, and Hep3B cell was homozygous deletion of p53 (Bhardwaj et al., 1999). In addition, the inhibition of proteasome and lysosome could not restore the decline of MARCH1 in Hep3B cells, suggesting that the degradation of MARCH1 in Hep3B cells was not through the proteasomal and lysosomal pathway. We found that BafA1 could inhibit HCC cells, and BafA1 with myricetin cotreatment further decreased MARCH1 expression. It is reported that BafA1 or CQ, combined with or without other medicine, could play a suppressive role on cancers (Hernández-Breijo et al., 2013; Balic et al., 2014; Lu et al., 2015). It was reported that CQ could decrease pancreatic cancer stem cells *via* inhibition of CXCL12/CXCR4 signaling (Balic et al., 2014). We found that CQ, as a autophagy inhibitor, decreased MARCH1 expression in the same way as BafA1. CQ cotreatment with myricetin could also further decrease MARCH1 expression. Therfore, further downregulation of MARCH1 may be one of the mechanisms of BafA1 or CQ synergistic anti-HCC with myricetin. Conversely, further downregulation of MARCH1 also proved that BafA1 and myricetin have synergistic anti-HCC effects. In addition, proapoptotic protein Bcl-2 was significantly downregulated in HCC cells after being treated with myricetin and BafA1. These results showed that BafA1 has synergistic anti-HCC effect with myricetin.

Autophagy was the main modality in which intracellular proteins and damaged organelles were degraded to maintain cellular homeostasis (Yang and Klionsky, 2009). Studies suggested that autophagy is prosurvival or suppressive for the growth of cancers in an environment- and stress-dependent manner (Puissant et al., 2010; Yang et al., 2020). The present study demonstrated that myricetin facilitated the activation of HCC cell autophagy and inhibited HCC growth. Autophagy could be modulated by various signaling pathways, such as PI3K-mTOR, AMPK-LKB1, and Stat3 pathways (Puissant et al., 2010; Liu et al., 2017). We found that myricetin activate HCC cell autophagy due to MARCH1 downregulation. This study showed that myricetin could also reduce p-Stat3 expression. Inhibition of p-Stat3 could activate autophagy. It is known that accumulation of P62 suggests the autophagy inhibition. P62 decrease indicates autophagy activation. However, Stattic not only enhanced autophagy but also increased P62 expression. It must be stressed that P62 is a multifunctional factor engaged in a variety of cellular pathways, and its protein level could also be changed independent of autophagy (Nakaso et al., 2004; Bardag-Gorce et al., 2005). For example, studies proved that hydroxytyrosol could elevate mRNA and protein level of P62 in RPE cells, but it is not related to the inhibition of autophagy (Zou et al., 2012). Oxidative stress in RPE-enhanced autophagic flux also increased p62 protein expression (Song et al., 2017). In addition, a combination of Akt inhibitor (AZD5363) and β-catenin inhibitor (FH535) in transformed hepatocytes activated autophagy in conjunction with increased p62 protein expression (Patra et al., 2020). Therefore, P62 increase in HCC cells treated by Stattic is not dependent on autophagy. Inhibition of p-Stat3 could also downregulate the MARCH1 expression. Our study found that MARCH1 could positively regulate the Stat3 signaling pathway. To sum up, myricetin induced an interaction between MARCH1 and Stat3 to activate HCC cells autophagy.

The smooth progression of cell cycle is an essential condition of cell proliferation. Cell cycle was regulated by various cellular mechanisms and cellular biological behaviors, such as signaling pathways and autophagy (Zhang et al., 2010; Zheng et al., 2019). This study demonstrated myricetin blocked HCC cell cycle at the G2/M phase to inhibit HCC cell proliferation. This consequence may partially be due to autophagy regulation. Eduardo C. Filippi-Chiela et al. reported that resveratrol activated cellular autophagy and induced glioma cells arrested at the G2/M phase (Filippi-Chiela et al., 2011). CyclinD1 was selectively recruited by autophagosome which fused with lysosome and degraded CyclinD1 in HCC cells, and resulted in cell cycle arrest at the G1 phase to inhibit cell proliferation (Wu et al., 2018; Wu et al., 2019). Kai Zheng et al. summarized that autophagy could regulate cell cycle progression by degrading cell cycle–related proteins (Zheng et al., 2019). In the current study, CyclinD1, CyclinB1, and Bcl-2 might be the degradation substrates of myricetin-mediated autophagy, and downregulation of these proteins was responsible for the G2/M phase arrest. Previous studies had reported that MARCH1 can regulate diverse signaling pathways, such as Wnt/β-catenin, PI3K/AKT/β-catenin, and PTEN/AKT pathways to control cancer development (Meng et al., 2016; Xie et al., 2019b; Dai et al., 2020). This study found that MARCH1 could regulate the p38 MAPK and Stat3 signaling pathways. Some studies claimed that p38 MAPK and Stat3 signaling pathways could regulate cell cycle to control cancer cell proliferation. Inhibition of p-p38 MAPK partially abrogated G2/M phase arrest induced by 10-methoxy-9-nitrocamptothecin in A549 cells (Zhang et al., 2010). SiRNA-Stat3 treatment can prolong G1 phase to inhibit H22 tumor growth (Tian et al., 2012). Current research revealed that myricetin could block HCC cell cycle at G2/M phase to suppress HCC growth by downregulating the MARCH1/p38 MAPK/Stat3 signaling pathway.

Taken together, myricetin induces HCC cell autophagy and cell cycle arrest at G2/M phase by downregulating MARCH1 to further inhibit HCC proliferation. Targeting MARCH1 could be a novel strategy for the treatment of HCC. However, inhibition of proteasome and lysosome could not restore the decline of MARCH1 in Hep3B. The main mechanism responsible for myricetin-induced decrease of the MARCH1 protein level in Hep3B is not clear and needs further research.

## Data Availability

The original contributions presented in the study are included in the article/Supplementary Material; further inquiries can be directed to the corresponding authors.
